# The Metabolomic Response of Crucian Carp (*Carassius carassius*) to Anoxia and Reoxygenation Differs between Tissues and Hints at Uncharacterized Survival Strategies

**DOI:** 10.3390/metabo11070435

**Published:** 2021-07-01

**Authors:** Helge-Andre Dahl, Anette Johansen, Göran E. Nilsson, Sjannie Lefevre

**Affiliations:** Section for Physiology and Cell Biology, Department of Biosciences, University of Oslo, 0371 Oslo, Norway; h.a.dahl@ibv.uio.no (H.-A.D.); anette.johansen@ibv.uio.no (A.J.); g.e.nilsson@ibv.uio.no (G.E.N.)

**Keywords:** metabolomics, glycolysis, electron transport chain, fumarate, succinate, alternative electron acceptor, purine nucleotide cycle, energetics, urea/uricolysis

## Abstract

The anoxia-tolerant crucian carp (*Carassius carassius*) has been studied in detail for numerous years, with particular focus on unravelling the underlying physiological mechanisms of anoxia tolerance. However, relatively little work has been focused on what occurs beyond anoxia, and often the focus is a single organ or tissue type. In this study, we quantified more than 100 metabolites by capillary electrophoresis-mass spectrometry (CE-MS) in brain, heart, liver, and blood plasma from four experimental groups, being normoxic (control) fish, anoxia-exposed fish, and two groups that had been exposed to anoxia followed by reoxygenation for either 3 h or 24 h. The heart, which maintains cardiac output during anoxia, unexpectedly, was slower to recover compared to the brain and liver, mainly due to a slower return to control concentrations of the energy-carrying compounds ATP, GTP, and phosphocreatine. Crucian carp accumulated amino acids in most tissues, and also surprisingly high levels of succinate in all tissues investigated during anoxia. Purine catabolism was enhanced, leading to accumulation of uric acid during anoxia and increasing urea formation that continued into 24 h of reoxygenation. These tissue-specific differences in accumulation and distribution of the metabolites may indicate an intricate system of transport between tissues, opening for new avenues of investigation of possible mechanisms aimed at reducing the generation of reactive oxygen species (ROS) and resultant tissue damage during reoxygenation.

## 1. Introduction

Most vertebrates rely on a continuous access to oxygen to support aerobic ATP production, fueling most cellular processes. In the absence of oxygen, oxidative phosphorylation is stalled and the cell is left with anaerobic glycolysis to meet the required ATP demand. Glycolysis yields much less ATP than oxidative phosphorylation [1] and the organism must increase the glycolytic rate or depress the metabolic activity, or both, in order to maintain energy balance. Most vertebrates are unable to maintain cellular ATP levels through anaerobic glycolysis during anoxia, and the resultant fall in ATP will soon lead to a cellular and mitochondrial membrane depolarization, initiating a cascade of deadly processes as well as potential acidosis with increasing levels of lactate from anaerobic glycolysis [2,3]. Furthermore, the reintroduction of oxygen to an anoxic organism may lead to increased production of reactive oxygen species (ROS) due to increased leakage of electrons from the reactivated electron transport system [4]. Accumulation of ROS can eventually lead to cell death because of the severe oxidative damage occurring to DNA and proteins [5,6,7,8]. Taken together, this means that most animal model systems used to investigate hypoxic survival will show pathological responses to both oxygen depletion and reoxygenation. 

Many aquatic environments vary in oxygen levels, with hypoxic episodes differing in duration and also severity, and organisms that live in these environments have some degree of hypoxia tolerance [9]. The crucian carp (*Carassius carassius*) display one of the most extreme examples of hypoxia tolerance, having the extraordinary ability of tolerating anoxia for long periods as it overwinters in frozen-over lakes and ponds, without experiencing an imbalance between ATP demand and production [10]. The unique physiological features of the crucian carp enables studies of natural, non-pathological mechanisms to tolerate long-term anoxic events and the following reoxygenation. 

Members of the genus *Carassius* have a striking and unique ability to, during anoxia, convert lactate into ethanol, which then freely diffuses across the gill surface [11]. This allows for continued glycolytic ATP production during oxygen depletion while avoiding toxic lactic acid accumulation. The evolution of the ethanol producing pathway was made possible through a whole-genome duplication in an ancestral species [12]. The crucian carp seems to survive anoxia as long as its glycogen stores last, which can be up to months in the wild, due to them having probably the largest liver glycogen storage capacity amongst vertebrates [13]. This maintained activity is in stark contrast to other anoxia-tolerant species such as the freshwater turtle *Trachemys scripta* that shuts down many functions and are profoundly metabolically suppressed [14,15]. In fact, similarities between fish of the genus *Carassius* and other anoxia-tolerant species are few. However, the crucian carp appear to share most of the anoxia adaptations with its close relative the goldfish (*Carassius auratus*) although some of these may have been lost or reduced due to domestication [16,17].

So far, most studies on the crucian carp have been focused on specific mechanisms important for oxygen transport during hypoxia, and anaerobic ATP production during anoxia [10]. Less is known about the mechanisms that allow the crucian carp to tolerate the inevitable process of reoxygenation, and how such responses may vary between tissues. For example, both the heart and the brain are highly energy-demanding tissues, and could be expected to display similar responses, but on the other hand, they have different cell types and structure, serving very different functions. While the brain further reduces its ATP demand during anoxia [18,19], beyond that which occurs as a result of temperature effects [20], the heart maintains cardiac output [21] and presumably energy demand. The liver is an important organ since it holds most of the glycogen necessary to survive prolonged anoxia, but the exact function and response of the liver in crucian carp exposed to anoxia-reoxygenation has rarely been in focus. In this study, we take a high-throughput approach to comprehensively investigate the metabolic events that occur not only when oxygen is depleted, but also when oxygen is reintroduced, the latter remaining an understudied aspect of crucian carp biology. Even though some studies on certain metabolites in the crucian carp have been conducted previously, many have focused on a rather limited set of metabolites or on single tissues [22,23]. Since crucian carp clearly exhibit extraordinary, effective mechanisms for anoxic survival that may not be paralleled in other animals, we find that it is now time for a more comprehensive and discovery-driven approach, including several organs, to elucidate the metabolic pathways involved in its responses to anoxia and reoxygenation. 

## 2. Results

### 2.1. Tissue-Specific Metabolic Profile

To explore how the metabolome of crucian carp change with oxygen depletion and return, we exposed wild-caught crucian carp to five days normoxia (normal oxygen levels), five days anoxia (<0.1% of air saturation) and five days anoxia followed by 3 h and 24 h reoxygenation. Analysis by capillary electrophoresis-mass spectrometry (CE-MS) targeting 116 metabolites (“C-scope”) was performed by Human Metabolome Technologies (HMT) on brain, heart, liver, and plasma. Differences between treatment groups were assessed with one-way ANOVAs followed by Tukey’s post-hoc tests, using the MetaboAnalyst online software [24,25]. Raw data from CE-MS analysis as well as fold changes, *p*-values and false discovery rates (FDR) for all metabolites are available in the electronic supplementary material (File S1).

**Figure 1:**
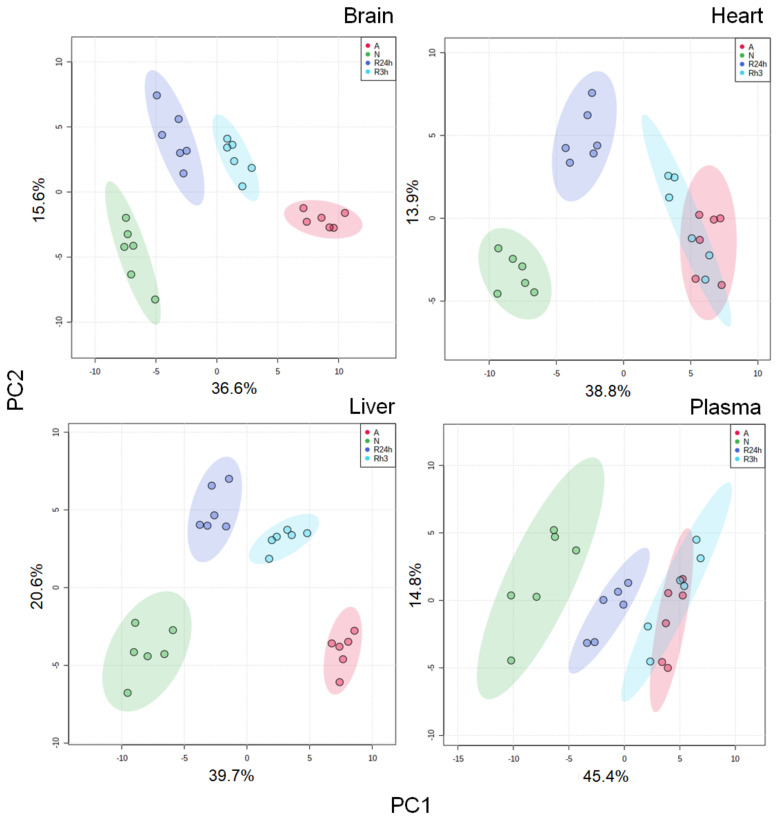
PCA plots. PCA plots for brain, heart, liver, and blood plasma for all exposure groups. A, anoxia; N, normoxia (control); R3h, 3 h reoxygenation; R24h, 24 h reoxygenation. PC1 and PC2: Principal component 1 and principal component 2. Each point represents a metabolite profile of a biological replicate (*n* = 6 for each group). The shaded area indicates the 95% confidence region.

Principal component analysis (PCA) showed that the investigated tissues responded in a similar manner to oxygen availability, although there are some striking differences related to the timing of the responses (Figure 1). The brain and liver metabolomes for each of the four treatment groups are distinctly grouped, with the normoxic and anoxic groups appearing to be the furthest apart. However, in the heart and blood-plasma metabolomes the anoxic group and the 3 h reoxygenation group are overlapping. The 3 h and 24 h reoxygenation are clearly different in their metabolic profiles in the tissues examined in this study, including blood plasma, indicating that key metabolites and pathways change over the course of reoxygenation. Overall, it seems like the heart begins recovery after the reintroduction of oxygen more slowly than the brain and liver, and that the plasma levels follow the same trend as the heart.

Exploring the data in detail, we found changes in metabolite levels related to the tricarboxylic acid (TCA) cycle, glycolysis, and purine degradation to be common to all investigated tissues. Amino acids and urea levels also changed with treatment. Succinate and uric acid levels were seen to be significantly changed in all tissues. In the following sections we will explore in more detail the changes involved in the processes mentioned above.

### 2.2. Purine Nucleotides and Energy State

**Figure 2:**
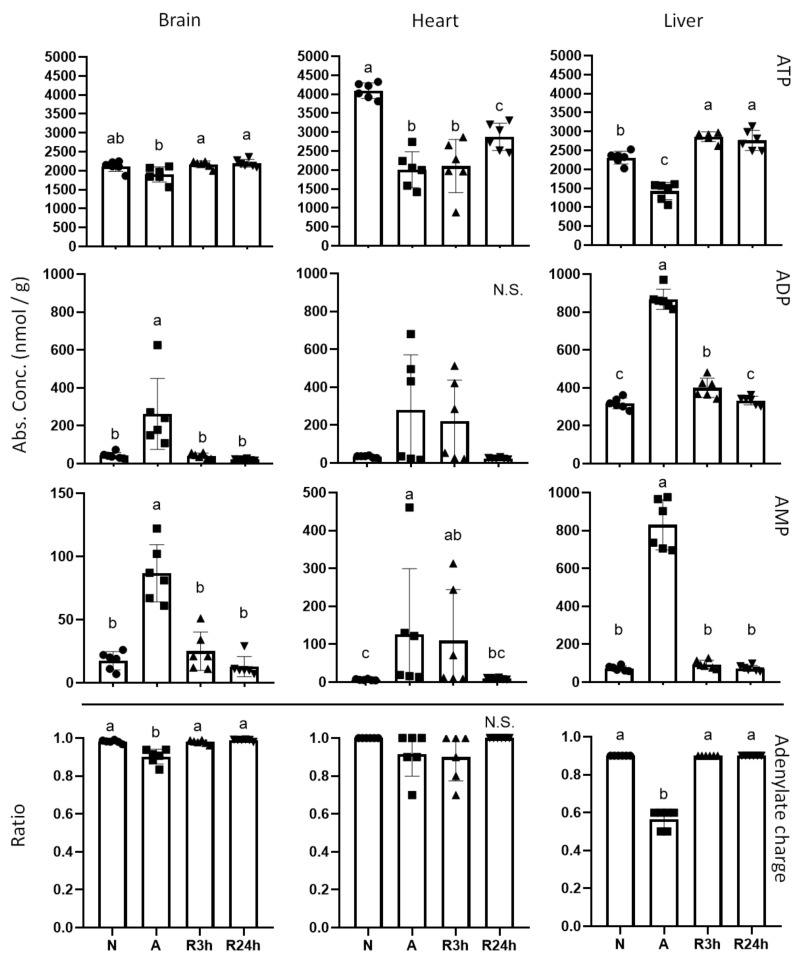
Adenosine phosphates. Graphs of absolute concentrations for ATP, ADP, AMP of brain, heart, and liver, as well as adenylate charges ([ATP + 1/2ADP]/[ATP + ADP + AMP]). N, normoxia (control); A, anoxia; R3h, 3 h reoxygenation, R24h, 24 h reoxygenation. Different letters indicate statistical differences between groups calculated using one-way ANOVA with Tukey’s post-hoc (*p*-value ≤ 0.05). Data are presented as means ± S.D. Values as reported by HMT (Human Metabolome Technologies).

**Figure 3:**
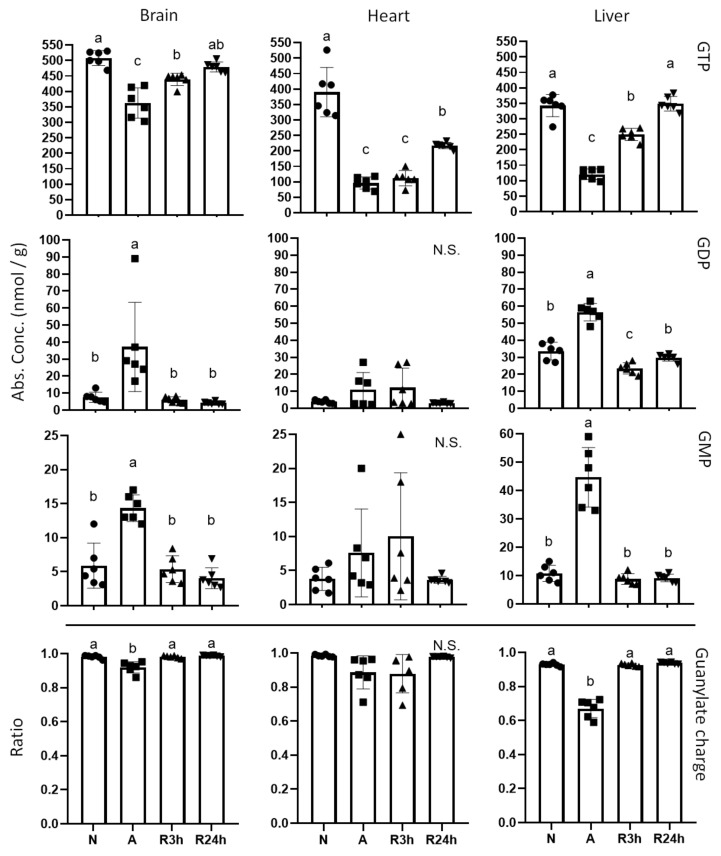
Guanosine phosphates. Graphs of absolute concentrations for GTP, GDP, and GMP of brain, heart, and liver, as well as guanylate charges ([GTP + 1/2GDP]/[GTP + GDP + GMP]). N, normoxia (control); A, anoxia; R3h, 3 h reoxygenation, R24h, 24 h reoxygenation. Different letters indicate statistical differences between groups calculated using one-way ANOVA with Tukey’s post-hoc (*p*-value ≤ 0.05). Data are presented as means ± S.D. Values as reported by HMT (Human Metabolome Technologies).

The largest differences between the 3 h and 24 h reoxygenation groups appear to be linked to the energetic state of the tissues as indicated by the adenylate and guanylate pools (Figure 2 and Figure 3). While the brain and liver seem to be recovering already after 3 h of reoxygenation, the same is not true for the heart. Of the three examined tissues, the anoxic heart experiences the greatest drop in ATP (Figure 2), around a 50% reduction, and the ATP level remains low even 3 h after oxygen has become available. Both brain and liver fully recovered to their normoxic ATP levels after 3 h of reoxygenation, whereas the heart is still recovering even after 24 h of reoxygenation. 

The equilibrium between the adenylates ATP, ADP, and AMP (i.e. adenylate charge) is indicative of the total energy state of a cell [26]. Despite the fact that ATP levels in all tissues are reduced in anoxia (and despite the lack of full recovery for the reoxygenated heart) the adenylate charge remained at relatively normal levels (Figure 2). Only the liver experienced a substantial and significant reduction in the adenylate charge, while the brain showed a very small but still significant drop, which could be detected due to the very small inter-individual variation within the groups. The adenylate charge appears to be slightly decreased also for the heart, but the inter-individual variation was larger than in the brain and the apparent change was not significant. 

While GTP is present at much lower concentrations than ATP, it also contributes to the energy transfer in cells, but with slightly different roles. GTP followed the same decreasing trends during anoxia as ATP (Figure 3), but the recovery time appears to be longer for GTP than ATP as the brain and liver are still not fully recovered by 3 h reoxygenation, while in the heart the GTP concentrations appear to remain at anoxic levels after 3 h of reoxygenation. By 24 h of reoxygenation the brain and liver GTP concentrations have returned to normoxic control levels while the heart has begun to recover. The guanylate charges appear to follow the same trends as the adenylate charges for each tissue type.

**Figure 4:**
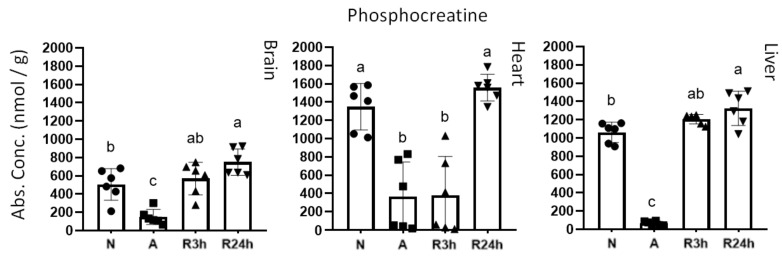
Phosphocreatine. Graphs of absolute concentrations for phosphocreatine of brain, heart, and liver. N, normoxia (control); A, anoxia; R3h, 3 h reoxygenation; R24h, 24 h reoxygenation. Different letters indicate statistical differences between groups calculated using one-way ANOVA with Tukey’s post-hoc (*p*-value ≤ 0.05). Data are presented as means ± S.D. Values as reported by HMT.

Phosphocreatine levels may, in addition to adenylate and guanylate charges, reflect the energetic status of tissues. Phosphocreatine functions as an immediate energy reserve that allows for the rapid regeneration of ATP from ADP via the donation of a phosphate group. The phosphocreatine levels exhibited similar recovery trends as ATP (Figure 4). In the brain and liver, phosphocreatine had returned to normoxic levels within 3 h of oxygen availability, indicating that there is a surplus of ATP allowing for the regeneration of phosphocreatine from creatine. In contrast, the phosphocreatine levels in the heart remained low both in anoxia and the 3 h reoxygenation groups, increasing to normoxic levels by 24 h of reoxygenation.

### 2.3. Glycolysis

**Figure 5:**
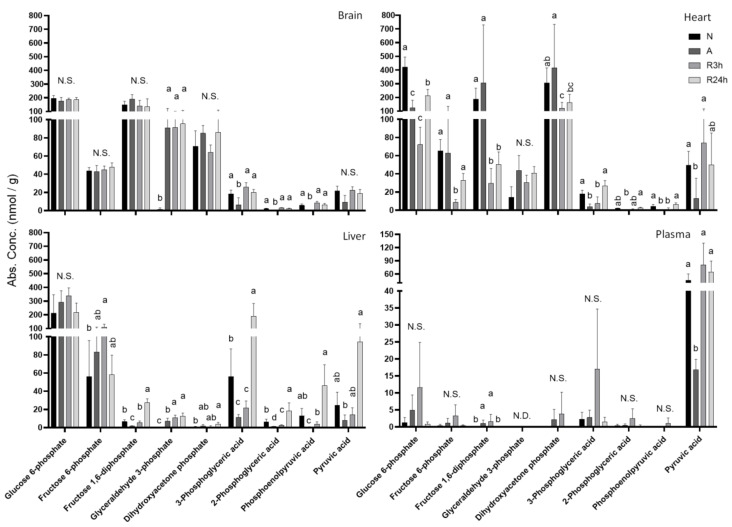
Glycolysis. Graphs of absolute concentrations of detected metabolites involved in glycolysis for brain, heart, and liver. N, normoxia (control); A, anoxia; R3h, 3 h reoxygenation; R24h, 24 h reoxygenation. Different letters indicate statistical differences between groups calculated using one-way ANOVA with Tukey’s post-hoc (*p*-value ≤ 0.05). Data are presented as means ± S.D. Values as reported by HMT.

Several metabolites involved in glycolysis showed tissue-specific differences, in particular upstream of glyceraldehyde dehydrogenase (Figure 5); specifically fructose 6-phosphate (F6P), fructose 1,6-phosphate (F1,6P), and glyceraldehyde 3-phosphate (G3P). Of these three intermediates only G3P was significantly changed in the brain, with a 58-fold increase during anoxia. Both F6P and F1,6P decreased in the heart during reoxygenation, while G3P possibly showed a small (non-significant) increase in all groups compared to normoxia. Whereas F6P in the liver tended to increase slightly throughout the treatments, F1,6P decreased in anoxia by 76% compared to normoxia before increasing during reoxygenation and reaching a 4-fold higher concentration after 24 h reoxygenation compared to the normoxic control group. Normoxic G3P levels in the liver were just above detectable levels and increased throughout both anoxia and reoxygenation to about 10-fold after 24 h reoxygenation compared to normoxia.

**Figure 6:**
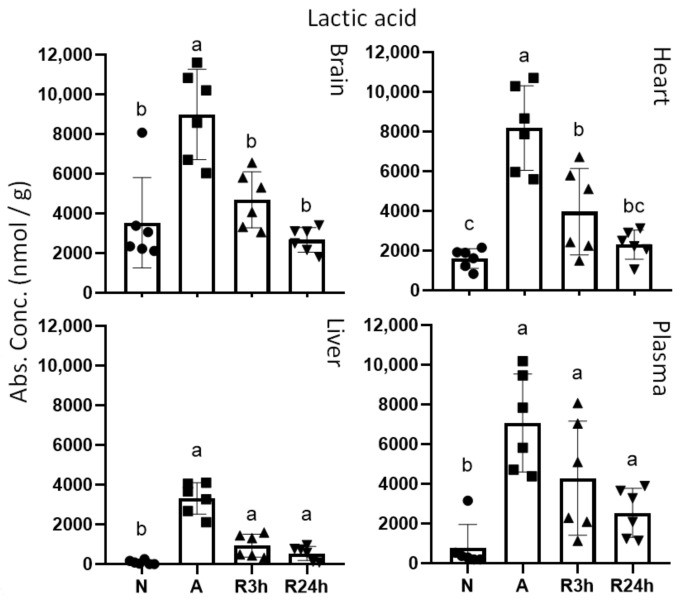
Lactic acid. Graphs of absolute concentrations for lactic acid of brain, heart, liver, and blood plasma. N, normoxia (control); A, anoxia; R3h, 3 h reoxygenation; R24h, 24 h reoxygenation. Different letters indicate statistical differences between groups calculated using one-way ANOVA with Tukey’s post-hoc (*p*-value ≤ 0.05). Data are presented as means ± S.D. Values as reported by HMT.

Lactic acid, generated through anaerobic glycolysis, accumulated substantially and displayed a similar pattern of change in all the anoxic tissues examined (Figure 6). Both brain and heart accumulated lactic acid in anoxia to approximately the same concentration, but the normoxic concentration was higher in the brain, which renders the fold increase in the brain to 3 and 5 in the heart. The concentration of lactic acid in anoxic liver was less than half of the other tissues, yet compared to the very low normoxic concentration the fold increase was the largest with 26. Interestingly, blood plasma showed an almost identical trend for lactic acid concentrations as the heart for all treatments, with a 9-fold increase from normoxia to anoxia. 

### 2.4. Tricarboxylic Acid Cycle

**Figure 7:**
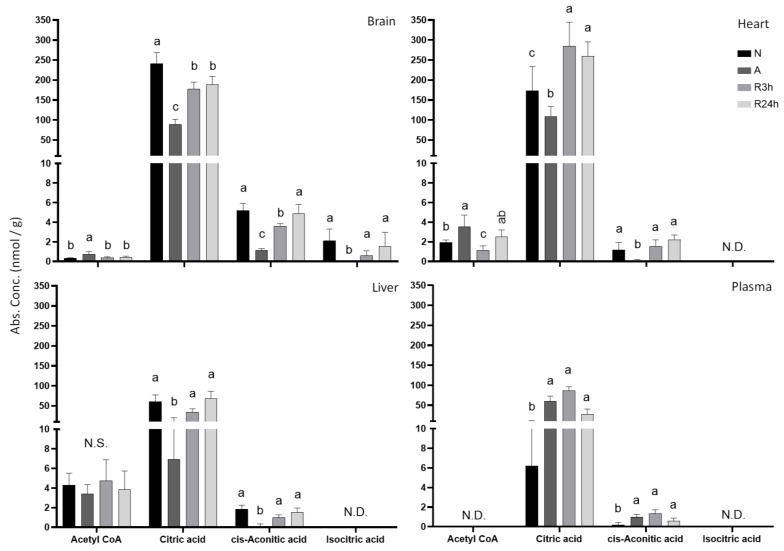
TCA cycle. Graphs of absolute concentrations for detected metabolites involved in the early stages of the TCA cycle of brain, heart, liver, and blood plasma. N, normoxia (control); A, anoxia; R3h, 3 h reoxygenation; R24h, 24 h reoxygenation. Different letters indicate statistical differences between groups calculated using one-way ANOVA with Tukey’s post-hoc (*p*-value ≤ 0.05). Data are presented as means ± S.D. Values as reported by HMT.

Acetyl-CoA that feeds into the tricarboxylic acid (TCA) cycle, as well as the first intermediates, citrate, and cis-aconitate, were all suppressed in the absence of oxygen in brain, heart, and liver, while increasing in the blood plasma although only found in low amounts (Figure 7). For all tissues except plasma, normoxic control levels were close to being reached within 3 h of reoxygenation, while the plasma levels were elevated further, and not returning closer to control levels before 24 h. Of the TCA cycle metabolites, alpha-ketoglutarate was not detected in more than half the samples of any tissues, while oxaloacetate was not measured for this study. Of the early cycle intermediates from acetyl-CoA to isocitric acid only citric acid was detected at levels over 8 nmol/g, and only citric acid and cis-aconitic acid were detected in more than 50% of the samples for all tissues. Isocitric acid was only detected in more than half the samples for the brain. Citric acid decreased in anoxia by approximately 63%, 37%, and 89% for the brain, heart, and liver, respectively, as compared to control. On the other hand, in blood plasma citric acid increased by about 10-fold during anoxia.

**Figure 8:**
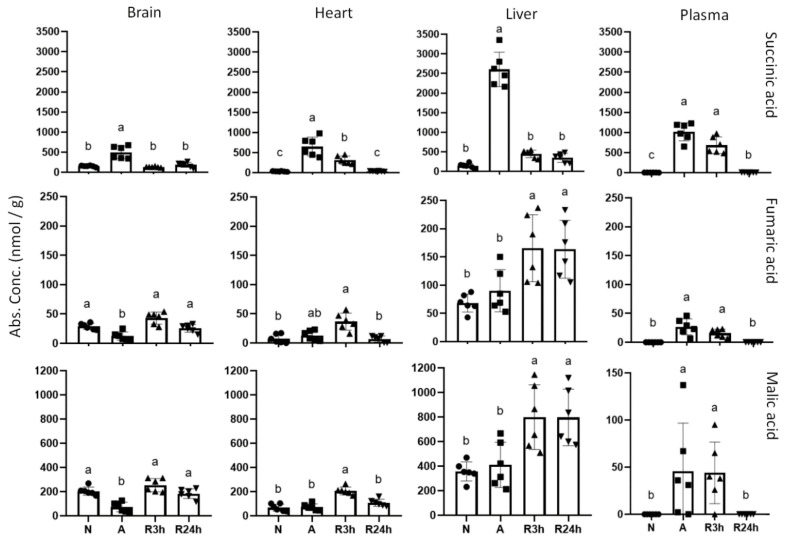
TCA cycle intermediates succinic acid, fumaric acid and malic acid. Graphs of absolute concentrations for succinic acid (succinate), fumaric acid (fumarate) and malic acid (malate) of brain, heart, liver, and blood plasma. N, normoxia (control); A, anoxia; R3h, 3 h reoxygenation; R24h, 24 h reoxygenation. Different letters indicate statistical differences between groups calculated using one-way ANOVA with Tukey’s post-hoc (*p*-value ≤ 0.05). Note that y-axis values are different. Data are presented as means ± S.D. Values as reported by HMT.

Interestingly, in later steps of the TCA cycle the metabolites showed stronger tissue-specific responses (Figure 8). While the metabolites of the first steps of the TCA cycles generally decreased except in blood plasma, succinate increased substantially, accumulating in all measured tissues and blood plasma during anoxia. The liver accumulated the highest concentration of succinate with an 18-fold increase, followed by blood plasma with 311-fold increase (going from 3 to 1000 nmol/g), then heart tissue with a 21-fold increase and finally the brain with a 3-fold change. After 3 h of reoxygenation succinate concentrations had returned to normoxic values in both brain and liver, while heart and plasma still displayed quite high succinate levels that were not recovered until 24 h reoxygenation. 

Malate and fumarate showed tissue-specific responses to the oxygen availability, although not as profoundly as succinate. Whereas fumarate and malate levels were decreased in the brain compared to the other experimental groups, we did not observe any changes in anoxic heart or liver whilst in plasma there seemed to be an increase in both anoxia and 3 h reoxygenation. We also observed an accumulation of at least malate in the heart at 3 h reoxygenation compared to the normoxic control and anoxic groups. A similar trend was also seen in the heart for fumarate, although the accumulation from anoxia to 3 h reoxygenation was not significant. Interestingly, malate and fumarate in the liver seemed to be accumulating mostly when oxygen was reintroduced. 

### 2.5. Amino Acids

**Figure 9:**
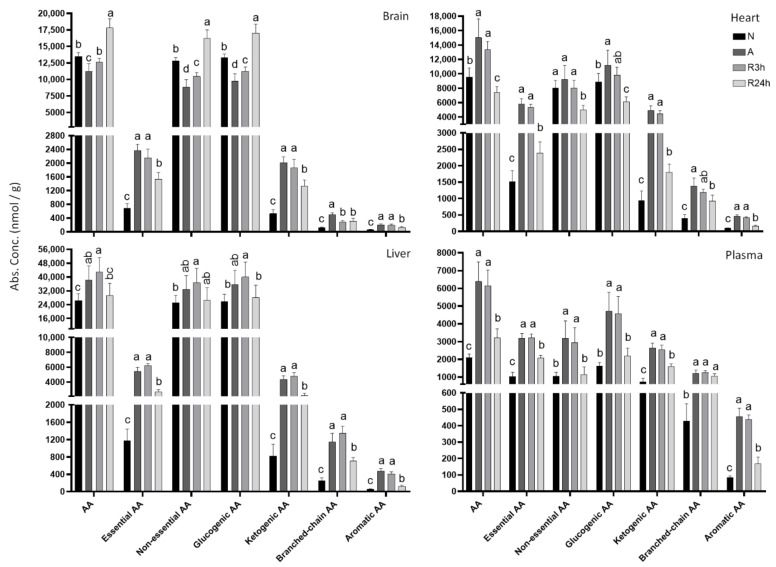
Amino acid totals. Graphs of absolute concentrations of total amino acids for brain, heart, liver, and blood plasma. N, normoxia (control); A, anoxia; R3h, 3 h reoxygenation; R24h, 24 h reoxygenation. Different letters indicate statistical differences between groups calculated using one-way ANOVA with Tukey’s post-hoc (*p*-value ≤ 0.05). Note that y-axis limits are different. Data are presented as means ± S.D. Values as reported by HMT.

At first glance, a general increase of total free amino acids was apparent in heart, liver, and blood plasma during anoxia, increasing by 1.6-, 1.5-, and 3-fold, respectively (Figure 9). This differed from the brain where the total free amino acid pool decreased slightly by about 17% in anoxia, and accumulated only after 24 h of reoxygenation. The distribution of the total free amino acids can be explained by the trends observed for the abundant non-essential ones (glycine, alanine, serine, proline, cysteine, asparagine, aspartate, glutamine, glutamate, tyrosine) and glucogenic ones (serine, proline, valine, threonine, cysteine, isoleucine, asparagine, glutamine, glutamate, methionine, histidine, phenylalanine, arginine, tyrosine, tryptophan), revealing that uniquely in the brain, these amino acids decreased in anoxia and accumulated to the highest concentrations after 24 h reoxygenation. The non-essential amino acids decreased by 31%, and the glucogenic amino acids by 27% in the anoxic brain. In contrast, the heart showed a slight increase of 1.5-fold during anoxia for non-essential amino acids, and a 1.3-fold increase for glucogenic amino acids. In anoxic liver, non-essential amino acids increased by 1.3-fold in anoxia, and glucogenic amino acids accumulated with a 1.4-fold increase. After 24 h reoxygenation the levels of non-essential and glucogenic amino acids were even lower than in normoxia in the heart, whereas in the liver they had returned to normoxic control levels. Overall, the plasma levels reflected the same trends, though with an even higher fold increase of total amino acids, non-essential, and glucogenic amino acids during anoxia and 3 h reoxygenation, than the heart and liver.

In common for all examined tissues, the essential amino acids in fish (valine, threonine, isoleucine, leucine, lysine, methionine, histidine, phenylalanine, tryptophan, arginine; based on [27]) increased substantially during anoxia and 3 h of reoxygenation. The essential amino acids increased by 3.5-fold in brain, 3.7-fold in heart, 4.5-fold in liver, and 3.2-fold in plasma. Even though the concentrations were much lower after 24 h of reoxygenation the levels had still not returned to normoxic values. Similar to the essential amino acids, the same trend was observed for ketogenic amino acids (threonine, isoleucine, leucine, lysine, phenylalanine, tyrosine, tryptophan), branched-chain amino acids (valine, isoleucine, leucine) and aromatic amino acids (phenylalanine, tyrosine, tryptophan) for all four tissues. 

**Figure 10:**
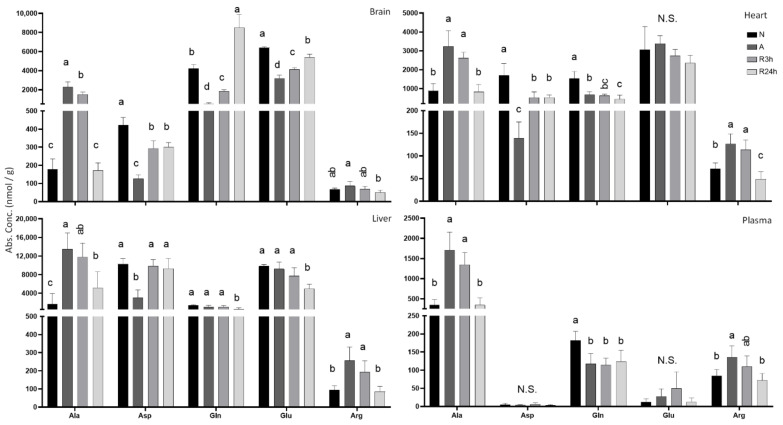
Amino acids of particular interest. Graphs of absolute concentrations of detected amino acids alanine, aspartate, glutamine, glutamate, and arginine for brain, heart, liver, and blood plasma. N, normoxia (control); A, anoxia; R3h, 3 h reoxygenation; R24h, 24 h reoxygenation. Different letters indicate statistical differences between groups calculated using one-way ANOVA with Tukey’s post-hoc (*p*-value ≤ 0.05). Note that y-axis limits are different. Data are presented as means ± S.D. Values as reported by HMT.

Glutamine, glutamate, and aspartate decreased substantially and enough to account for the decrease in total amino acids in the brain during anoxia with approximately 88%, 50%, and 70% decreases respectively (Figure 10). Furthermore, aspartate was also decreased by 70% in anoxic liver and 92% in the anoxic heart, whereas glutamine levels decreased by 56% in the anoxic heart compared to normoxia. Alanine accumulated during anoxia and 3 h reoxygenation in all studied tissues, increasing by 4-fold in heart, 8-fold in liver and 12-fold in brain in anoxia compared to control normoxia. Arginine increased in all studied tissues during anoxia, and started recovery after 3 h of reoxygenation, reaching control levels by 24 h reoxygenation. The remaining detected amino acids can be found in Appendix A Figure A3.

### 2.6. Purine Nucleotide Catabolism

**Figure 11:**
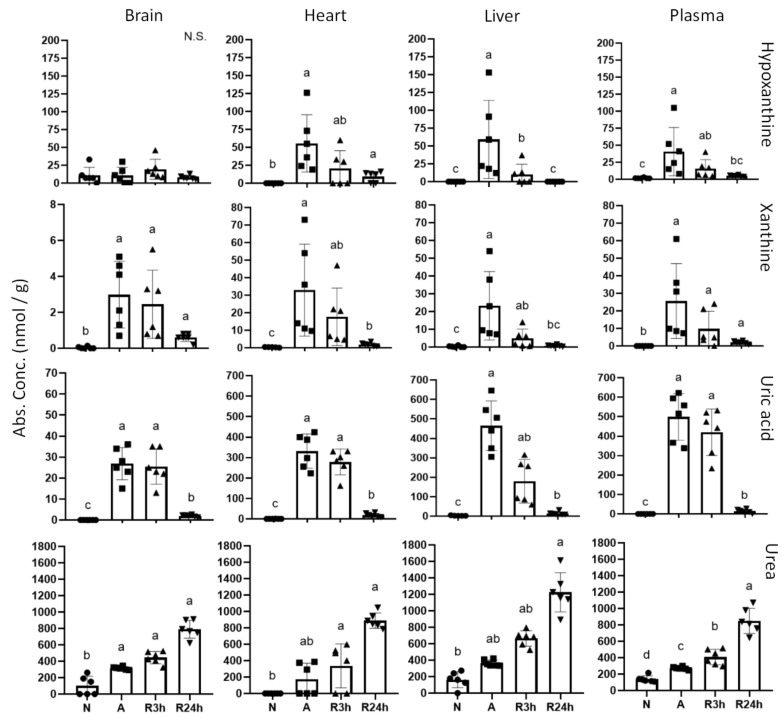
Purine metabolism. Graphs of absolute concentrations of metabolite intermediates hypoxanthine, xanthine and end-product uric acid, as well as urea for all tissues. N, normoxia (control); A, anoxia; R24h, 24 h reoxygenation; R3h, 3 h reoxygenation. Different letters indicate statistical differences between groups calculated using one-way ANOVA with Tukey’s post-hoc (*p*-value ≤ 0.05). Note that y-axis limits are different. Data are presented as means ± S.D. Values as reported by HMT.

The intermediates of the purine nucleotide catabolic pathway (hypoxanthine, xanthine, and uric acid) showed a tendency of accumulating during anoxia and recovering to normoxic values after 24 h reoxygenation (Figure 11). Hypoxanthine and xanthine showed a relatively small increase in terms of concentration; however uric acid increased more drastically. Uric acid was not detected in any tissue or plasma from the normoxic controls, but was increased in anoxia, most notably in liver and blood plasma. The lowest increase was in brain tissue, while heart saw an increase to approximately 300 nmol/g during anoxia, and the uric acid levels remained elevated at similar amounts even after 3 h of reoxygenation.

Even though levels were not significantly different between all treatment groups, urea concentrations increased substantially in all tissues, starting to accumulate in anoxia and continuing through both 3 h and 24 h of reoxygenation. Concentration in all tissues increased approximately 6- to 8-fold. The heart differs as no urea could be detected during normoxia, but increased to almost 900 nmol/g within 24 h of reoxygenation. Urea levels were highest in the liver, with a 7.5 fold increase from control levels after 24 h reoxygenation. The increase was almost linear from normoxia until 3 h of reoxygenation, with a slightly larger increase between 3 h and 24 h reoxygenation. 

## 3. Discussion

### 3.1. Energy-Carrying Compounds

Since most previous studies have focused on the effects of anoxia, or have had a single reoxygenation time-point of 24 h or longer, the differences in the timing of recovery responses has not previously been in focus. With the addition of the 3 h reoxygenation time point, we have not only found that the tissues respond differently to reoxygenation, but also that within each tissue there are differences in responses between the two reoxygenation time points. As revealed by the PCA plot (Figure 1), the two reoxygenation periods do not group for any of the tissues, indicating that tissue-specific processes are occurring during the course of reoxygenation. 

In addition, the heart and blood plasma differ in their recovery time, illustrated by the overlapping of 3 h of reoxygenation and anoxia and reflected in quite a few of the metabolites. With regards to the heart, recovery seems to be stalling upon reoxygenation, which is best reflected by the energetic status of the heart (Figure 2, Figure 3 and Figure 4). Neither ATP, GTP, nor phosphocreatine show a noticeable recovery by 3 h of reoxygenation, and are not even fully recovered by 24 h. In contrast, both brain and liver have fully recovered their stores of both ATP and phosphocreatine by 3 h of reoxygenation, and have started to recover their GTP levels. The recovery of phosphocreatine in the brain and liver indicates that the ATP levels were being restored before 3 h of reoxygenation had passed, as phosphocreatine replenishment is highly dependent on the energetic status of the tissues [28]. 

This finding that ATP in the heart is still at anoxic levels after 3 h reoxygenation is surprising. Oxidative phosphorylation is much more efficient in ATP generation than anaerobic glycolysis and we would have expected it to resume relatively quickly once oxygen is available and that consequently the level of ATP would increase. Since the crucian carp heart is composed of mostly spongy myocardium lacking coronary arteries [29] and therefore relies on oxygen supply from the venous blood flowing through its lumen, it may be that other tissues have taken up so much oxygen during the initial reoxygenation period that little is left for the heart. The brain and liver, which receives oxygenated arterial blood, may show an elevated rate of oxygen consumption aimed at rebuilding glycogen and phosphocreatine stores as well as oxidizing lactate. However, it is also possible that the recommencing of oxidative phosphorylation is strictly controlled in the heart in order to suppress the production of ROS and related cellular damage. 

The adenylate charge in anoxic heart and brain remained above the threshold of 0.7 to 0.95 that is considered to allow for anabolic reactions to progress [30], in fact they appear to stay close to 0.9 regardless of treatment. In contrast, liver adenylate charge was reduced to 0.6 during anoxia (Figure 2), which indicates that the anoxic liver may be within the amphibolic range, that is, stimulating both anabolic and catabolic processes [26,31]. The heart and brain maintenance of adenylate charge is likely achieved by the suppressed metabolic state and sufficient supply of anaerobically produced ATP, combined with the continued breakdown and recycling of ADP and AMP. The observed accumulation of the purine nucleotide intermediates xanthine, hypoxanthine and particularly the end product uric acid confirms that AMP, as well as GMP, is degraded during anoxia (Figure 11). Interestingly, whereas liver is the tissue with the most reduced adenylate charge, the heart experienced the largest drop in ATP. Our findings support that the liver degrades ADP and AMP more slowly than brain and heart, resulting in a larger accumulation of these nucleotides and therefore lower adenylate charge ratio. The same slow degradation is seen for liver GDP and GMP, and indeed the guanylate charge follows the same trend as the adenylate charge. 

### 3.2. Metabolites from Glycolysis and the TCA Cycle

#### 3.2.1. Lactate

During oxygen deprivation, oxidative phosphorylation ceases and pyruvate conversion to, and resulting accumulation of, lactate will in most species rapidly lead to lactic acidosis. The crucian carp’s special ability to transport lactate to red muscle tissues for conversion to ethanol, which is further released over the gills, enables it to avoid acidosis, and allows for a higher glycolytic flux to match the ATP demand [13,32,33]. The glycolytic metabolites included in this study showed tissue specific trends (Figure 5 and Figure 6) which may be explained by the differential energetic demands of each tissue. 

Anoxic heart accumulated the largest amount of lactate, probably reflecting its presumably higher energy demand relative to other organs, due to the maintained cardiac output. These results are in contrast to a previous study on crucian carp that did not observe any increase of lactate in the heart [33], whereas they only report a moderate lactate accumulation in the brain and a smaller increase in liver and blood plasma during anoxia. This difference may be attributed to the lower exposure temperature (2 °C in the mentioned study compared to 8 °C in this study) since temperature will likely reduce the metabolic rate further. In the present study, we show considerable lactate accumulations in all four tissues. Lactate accumulated in the liver to a lower concentration than in the other examined tissues during anoxia, which probably reflects a lower glycolytic rate compared to heart and brain. Glycogen breakdown is greatly enhanced in the liver when oxygen is depleted, but the produced glucose is predominately exported to other tissues [13,33]. As expected during anoxia, lactate levels increased in blood plasma, reflecting lactate transport from different tissues to the red muscle for conversion to ethanol. 

Both brain and heart lactate levels returned to normoxic control values after reoxygenation, while in the liver there was still more lactate present after 24 h reoxygenation than in normoxia. This tissue specific pattern of lactate clearance during reoxygenation may be explained either by that lactate produced in the liver is exported more slowly to the bloodstream than in brain and heart, or it may be that lactate is taken up by the liver. Since crucian carp start to build up the glycogen stores once oxygen is back [33], lactate imported to the liver could serve as a substrate for gluconeogenesis.

**Figure A1:**
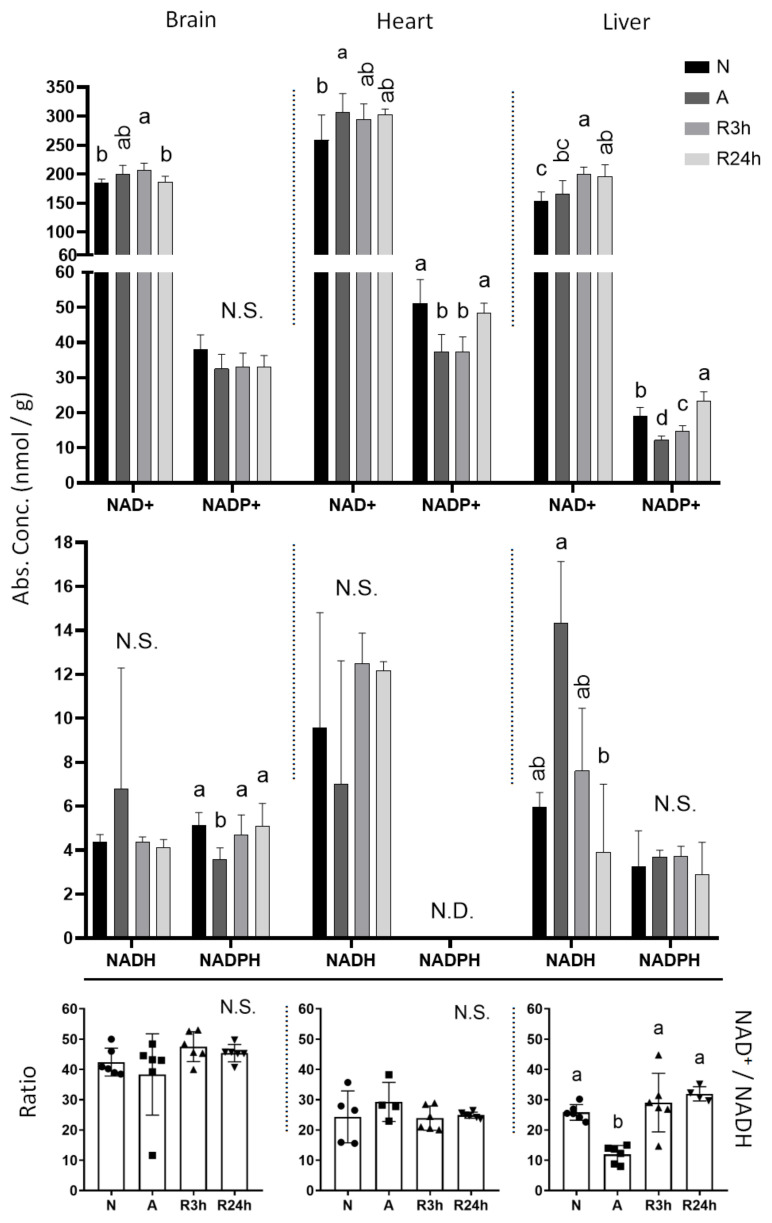
NAD^+^, NADH, NADP, NADPH, and NAD/NADH ratio. Graphs of absolute concentrations of NAD+, NADH, NADP, NADPH, and NAD^+^/NADH ratios for brain, heart, and liver. N, normoxia (control); A, anoxia; R3h, 3 h reoxygenation; R24h, 24 h reoxygenation. Different letters indicate statistical differences between groups calculated using one-way ANOVA with Tukey’s post-hoc (*p*-value ≤ 0.05). Data are presented as means ± S.D. Values as reported by HMT. Individuals in which either NAD^+^ or NADH was not detected were excluded from NAD^+^/NADH ratio calculation.

The generation of lactate is likely to be partially responsible for maintenance of NAD^+^ to NADH ratios, as has been seen in the freshwater turtle [34], alongside alternative fates for pyruvate and amino acids (discussed in Section 3.3). Indeed, we noted that NAD^+^ and NADH levels were relatively stable during all exposures (Appendix A Figure A1), perhaps with the exception for the liver in which the NAD^+^/NADH ratio drops slightly. Additionally, crucian carp as well as the western painted turtle (*Chrysemis picta bellini*) appear to reduce their expression of glyceraldehyde 3-phosphate dehydrogenase (GAPDH), which is likely a mechanism to conserve energy, as well as potentially functioning as an anti-apoptotic mechanism [35,36]. This reduction of GAPDH levels in the brain may explain the high accumulation of G3P in the brain, as it limits the rate of G3P progression through the ensuing glycolytic stages. G3P can function as an electron donor in the glycerol phosphate shuttle (GPS), a pathway similar to the malate–aspartate shuttle (MAS). In the GPS, cytosolic glycerol 3-phosphate dehydrogenase (GPDH) will regenerate NAD^+^ through the conversion of DHAP to G3P. However, this can also function in reverse, as G3P conversion to DHAP is possible through the mitochondrial GPDH, and in this direction, it aids in the maintenance of the mitochondrial membrane potential [37]. Whereas G3P accumulated substantially in the brain, the other glycolytic intermediates prior to GAPDH appear to remain stable, including DHAP which saw a relatively small but insignificant increase, and thus it is uncertain to what degree G3P/DHAP interconversion occurs in order to maintain NAD^+^/NADH ratios or mitochondrial membrane potential.

#### 3.2.2. Succinate

The TCA cycle releases the energy of acetyl-CoA mainly in the form of NADH, which can feed electrons into the electron transport chain (ETC) of mitochondria, ultimately for the formation of ATP. Under anaerobic conditions, however, the final electron acceptor (oxygen) in ETC is no longer present, leading to altered fates of the involved metabolites [38]. One such metabolite that has been of increasing interest in studies of ischemia-reperfusion injury is succinate, which is frequently and consistently increased in ischemic tissues and accepted as a hallmark of ischemia [39,40]. It is proposed that this increase is a result of succinate dehydrogenase (SDH) reversal in order to aid in maintaining the mitochondrial membrane potential [41,42]. Succinate may additionally be accumulating through the anaerobic metabolism of glutamate and aspartate (discussed in next section) [43]. Succinate accumulation in crucian carp during anoxia has been noted previously in heart tissue by our research group (Scott et al., unpublished), and in goldfish muscle, liver, and blood [38]. In the present study we find increased succinate levels during anoxia in all the tissues studied including blood plasma (Figure 8), suggesting a global response to anoxia.

Despite the fact that the crucian carp does not experience ischemia during anoxia, the levels of succinate accumulated in the brain and heart are similar to those of anoxia intolerant species undergoing ischemia at low temperatures [44]. However, crucian carp liver and blood plasma accumulated considerably more succinate than heart and brain. In murines, the liver accumulates more succinate than other tissues during ischemia [41], in line with the high succinate levels found in anoxic liver in this study.

Succinate decreased within 3 h of reoxygenation while the down-stream metabolites fumarate and malate increased compared to anoxia, indicating that TCA cycle activity recovers early during reoxygenation. The rapid oxidation of succinate by SDH in the normal (i.e., aerobic) forward direction has been postulated to lead to reverse electron transport to mitochondrial complex I and resulting in the generation of ROS [41]. Furthermore, succinate efflux from the heart [45,46,47] can decrease ROS production upon reoxygenation [41]. Taking into account the greatly elevated liver and plasma levels of succinate in crucian carp during anoxia, we speculate if succinate is transported to the liver to limit the production of ROS during reoxygenation in other tissues. Since the liver has high capacity for regeneration [48] and contains high levels of antioxidants [49], it could be the best suited tissue for handling ROS, and may serve the whole body as a site for succinate processing. It has also previously been seen in goldfish that the liver experiences the most oxidative damage as compared to kidney, brain, and white muscle, as well as increased catalase and glutathione reductase activity after anoxia, and having high constitutive levels of antioxidants [50,51]. Along the same lines of thought, it is interesting to note that the crucian carp liver showed the highest accumulation of uric acid, which may function as an antioxidant [51]. 

### 3.3. Amino Acids

A general trend noted in the metabolomics data was the accumulation of free amino acids. In contrast, free amino acids decrease in the heart of anoxia-tolerant turtles when oxygen is unavailable [14]. The differential abundance of amino acids has been observed to some extent in crucian carp earlier [13,22], yet this was only investigated on a selection of less than 10 amino acids, and conducted on fish subjected to different exposure times than in the present study. Our findings confirm the previously reported observations on amino acid metabolism, including decreased levels of glutamine, glutamate, and aspartate and increased levels of alanine and glycine in the anoxic crucian carp, and the decreased levels of aspartate in the goldfish liver during anoxia [38]. Recovery towards normoxic levels was relatively slow for many of the amino acids, which may be explained by slow recovery of normal cellular activities (such as protein synthesis), slow metabolism of the amino acids, or a potential role during reoxygenation. Amino acids have also been suggested to be used for resynthesis of glycogen in crucian carp [33].

**Figure A2:**
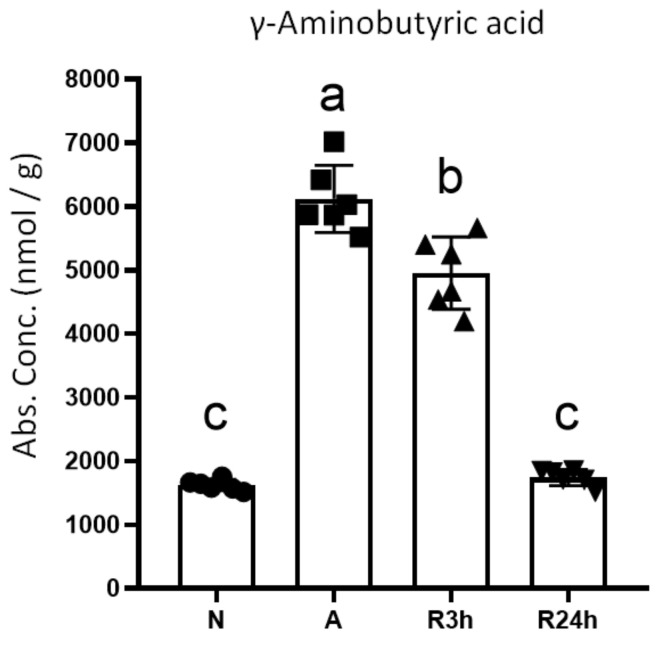
γ-aminobutyric acid from brain tissue. Graphs of absolute concentrations for γ-aminobutyric acid (GABA) of brain. N, normoxia (control); A, anoxia; R3h, 3 h reoxygenation; R24h, 24 h reoxygenation. Different letters indicate statistical differences between groups calculated using one-way ANOVA with Tukey’s post-hoc (*p*-value ≤ 0.05). Data are presented as means ± S.D. Values as reported by HMT.

**Figure A3:**
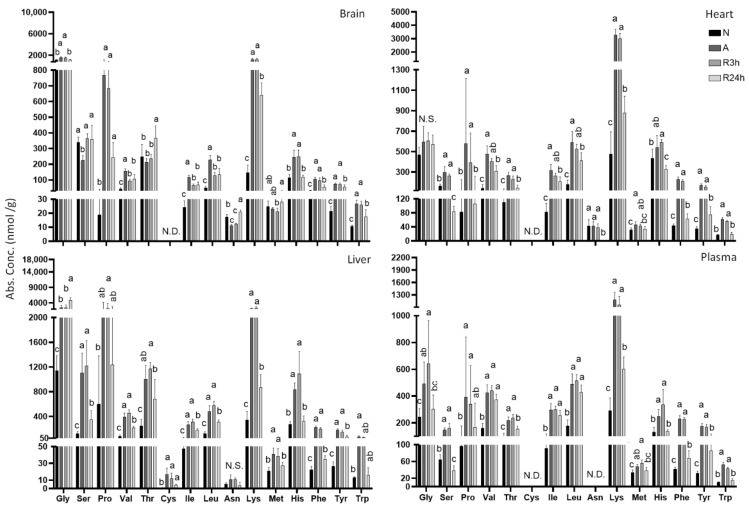
Amino acids. Graphs of absolute concentrations for amino acids of brain, heart, liver, and blood plasma, excluding the amino acids in Figure 10. N, normoxia (control); A, anoxia; R3h, 3 h reoxygenation; R24h, 24 h reoxygenation. Different letters indicate statistical differences between groups calculated using one-way ANOVA with Tukey’s post-hoc (*p*-value ≤ 0.05). Data are presented as means ± S.D. Values as reported by HMT.

The overall similar trend for the amino acids regardless of tissue type suggests that the amino acids generally serve the same purposes, with a few exceptions such as glutamine in the brain that decreased more than in the other tissues. Glutamine and glutamate are both involved in the generation of the neurotransmitter GABA, known to increase in the anoxic crucian carp brain [18]. In our data the GABA concentration increased 3-fold in the anoxic brain compared to normoxic values (Appendix A Figure A2), and may result in the lack of a general increase of amino acids in the brain during anoxia as was observed in the other tissues and blood plasma. However, glutamine levels decreased in heart and to some extent in plasma, indicating additional roles. Aspartate displayed relatively large decreases in anoxia, probably caused by its involvement in the aspartate-succinate pathway (particularly in the heart and liver), or gluconeogenesis in the liver. Both of these pathways may have a role in anoxia, be it maintenance of membrane potential, glucose synthesis, or alternate ATP generation; the latter scenario being discussed below. 

**Figure 12:**
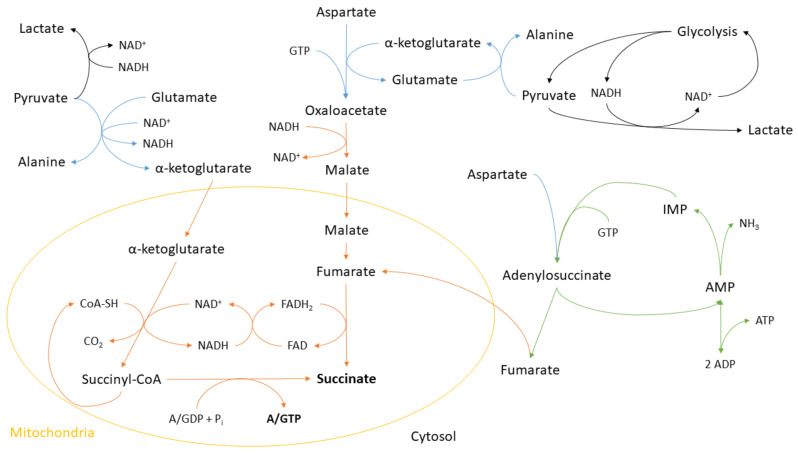
Succinate generation during anoxia. Anoxic succinate accumulation related to amino acid metabolism (blue arrows), glycolysis (black arrows), and purine nucleotide cycle (green arrows). Left: Transamination of pyruvate from glycolysis to alanine generates alpha-ketoglutarate from glutamate. Alpha-ketoglutarate can then be fueled into the TCA cycle, producing ATP/GTP via succinyl-CoA conversion to succinate. Middle: Alpha-ketoglutarate may also drive the formation of oxaloacetate from aspartate. Through reverse-functioning TCA cycle, malate is converted to fumarate and succinate, generating ATP and regenerating NAD^+^. Right: Aspartate can also contribute to fumarate generation in the purine nucleotide cycle.

Increasing alanine levels have been observed in both goldfish and crucian carp previously [52,53,54]. It may in part be due to the transamination of pyruvate from glycolysis to alanine, a mechanism used to generate alpha-ketoglutarate from glutamate (Figure 12). Alpha-ketoglutarate may be further processed in the TCA cycle to generate NADH and succinyl-CoA, leading to ATP/GTP generating hydrolysis of succinyl-CoA into succinate as an end product. In ischemic mice hearts, succinyl-CoA has been suggested to be the main contributor to succinate accumulation [45], and notably, succinate-CoA ligase transcription increased in the liver of anoxic turtles [55]. 

Alpha-ketoglutarate may also drive the formation of oxaloacetate from aspartate (Figure 12). NADH produced from glycolysis can then be used to reduce oxaloacetate to malate, regenerating NAD. Through the malate–aspartate shuttle [56], malate can enter mitochondria and be converted to fumarate in a reverse-functioning TCA cycle which is thought to contribute to succinate formation [39,41]. However, there are certain pathways via citric acid which may circumvent the malate–aspartate shuttle, perhaps further complicating the system [56]. At the same time, NADH generated in the succinyl-CoA producing step would be oxidized back to NAD. Aspartate may also contribute to succinate production via the purine nucleotide cycle (Figure 12). Inosine monophosphate (IMP) isconverted to AMP in a manner that releases fumarate, which in the case of the liver may be a reason for the relatively high levels of AMP and ADP as opposed to other tissues. 

The formation of succinate using either aspartate or glutamate seems reasonable, since this would lead to additional energy sources to supplement anaerobic glycolysis at the same time as reducing equivalents are kept in balance. Particularly aspartate to succinate via oxaloacetate appears beneficial since glutamate would be regenerated in a cyclical manner. This scenario would be in line with the observed unchanged glutamate levels in heart and liver, suggesting that glutamate-to-succinate conversion may be occurring to a smaller extent. However, the maintained glutamate levels may also result from deamination of glutamine to form glutamate. Indeed, the glutamine levels decrease quite substantially in the brain and heart, but to a far lesser degree in the liver. These possible pathways leading to succinate have been discussed previously in detail in regards to goldfish [38], anaerobe diving vertebrates [57], turtles [14], and ischemia-reperfusion in anoxia intolerant species [39,41]. 

Since crucian carp do not feed (and were not fed) during anoxia, and thus were not receiving any amino acids via their diet, it is most likely that the overall increase in amino acids is the result of protein degradation. In goldfish, there has been evidence towards proteolysis being a secondary source of fuel used in addition to glycogen [38,52], in which alanine is further fermented and released as ethanol and ammonia [38]. Amino acids from proteolysis may also be supplied to the tissues via the blood from skeletal muscles [58,59]. Additionally, alanine may be transported to the liver, where it has been suggested to act as a precursor to gluconeogenesis [58,60]. Interestingly, large alanine accumulation in liver tissue has also been seen in the Siberian wood frog *Rana amurensis* during extreme hypoxia [61], indicating that alanine may be part of a common mechanism for anoxia-tolerant as well as hypoxia-tolerant species. This additional source of energy would potentially reduce the rate of glycolysis and in this way conserve glucose, which would benefit the crucian carp greatly during prolonged anoxia exposures.

### 3.4. Purine Metabolism

In order to maintain adenylate charge, we expected to find accumulating AMP to be degraded, especially in the liver. Indeed, the increasing levels of hypoxanthine, xanthine and uric acid do support that AMP is degraded by the purine nucleotide degradation pathway during anoxia, with the highest uric acid concentrations in the liver and the heart. The xanthine oxidoreductase responsible for the oxidation of hypoxanthine and xanthine can utilize both O_2_ and NAD^+^, obviously using the latter during anoxia [62]. Further processing of uric acid to urea (i.e., uricolysis) by the enzyme uricase requires oxygen [63]. We observed no decrease in uric acid levels after 3 h of reoxygenation in any of the investigated tissues, perhaps with the exception of a non-significant decrease in liver. Clearly more than 3 h of reoxygenation is required for uric acid to return to normoxic levels, whereas degradation in the liver would be in accordance with the presence of hepatic uricase in many freshwater teleosts [64].

It was surprising to find that uric acid increased most in liver and plasma, with both tissues accumulating it to the same concentration. Since the liver seems to have a slower breakdown of ADP and AMP, in addition to a lower decrease in ATP levels, we did not expect the liver to have the largest accumulation of uric acid. The high levels of uric acid in the plasma indicate that it is being transported, perhaps from other tissues to the liver, in order to be converted to urea. 

Multiple pathways may lead to the formation of urea in fish, such as uricolysis (purine nucleotide breakdown), ornithine-urea cycle (OUC), and arginolysis [65]. In most teleosts the majority of ammonia is excreted without the formation of urea. Even though some ammonotelic teleosts seem to retain enzymes for the OUC, these are not actively used, at least not in adult life stages [66]. Additionally, a study on goldfish did not detect any activity or expression of the OUC-exclusive enzymes [67]. Another report on crucian carp exposed to high ammonia levels showed that the plasma urea concentration remained constant during the exposure, indicating that ammonia is not converted to urea [68]. Altogether, crucian carp seem to be similar to other ammonotelic teleosts in that urea is mainly formed using either arginolysis or uricolysis [65,69]. 

Intriguingly, and in contrast to the heart and liver, the accumulating urea levels in the brain cannot be explained solely by formation from uric acid. Each uric acid molecule is processed to give two urea molecules, but the urea level in the brain is more than 10 times higher than the uric acid concentration (Figure 11). Combined with the insignificant decrease of ATP during anoxia in the brain, it is clear that uricolysis alone is not the only urea producing pathway. Since the OUC is not the likely route, arginolysis could be a possible pathway for urea production. Arginase in the brain is known to be responsible for the contribution of glutamate through arginine conversion to ornithine and the subsequent γ-aminobutyric acid (GABA) production [70]. However, arginine levels in the brain remain similar to the normoxic conditions (Figure 10). Aspartate can contribute to maintaining the arginine pool in other vertebrates, but in fish arginine is an essential amino acid [27,71]. In addition, the largest decrease in aspartate concentrations is observed in the heart, and not the brain (Figure 10). It is presently unclear why there is an increase in urea from approximately 100 nmol/g in normoxia to approximately 800 nmol/g by 24 h of reoxygenation in the brain. 

Our data do not reveal if the urea concentration peaks after 24 h of reoxygenation or if it continues to increase further. Urea seems to start accumulating already during anoxia also in the heart and liver, which is hard to explain by the oxygen-dependent conversion of uric acid to urea. In contrast to the brain, arginine is accumulating in the anoxic heart and liver (Figure 10). Even though arginolysis mainly occurs in the liver [65], it is difficult to explain the urea build-up in the other tissues based on the rather modest increase of arginine in the liver, especially in terms of the substantial urea accumulation in the brain.

Clearly, the brain copes with anoxia differently than the heart and liver, also in terms of urea production. It is puzzling where the urea comes from and what function, if any, it might serve. Perhaps there is some form of active uptake of urea to the brain in order to balance the high plasma urea levels that could otherwise cause osmotic forces to move water out from the brain across the blood–brain barrier. Indeed, the crucian carp brain seems to accumulate water and swell during anoxia [68], and several other fishes and amphibians have been shown to prevent water loss by producing and retaining urea [72]. We can only speculate what the exact purpose of urea is in terms of anoxia tolerance in crucian carp. Urea is involved in maintenance of acid–base balance and osmolarity regulation, and in certain species of marine fish it may act as a positive buoyancy solute [73]. The freeze- and anoxia tolerant wood frog *Rana sylvatica* use both urea and glucose as cryoprotectants to avoid freeze–thaw damage [74,75], a possible mechanism for the crucian carp surviving in shallow ponds that freeze through to the bottom. Indeed, crucian carp have been found to occasionally be frozen in lumps of ice or into the mud while still surviving [33]. Additionally, while urea-induced ROS production has been reported in chronic renal failure in humans [76], urea has interestingly been proposed to exhibit antioxidant protection during cardiac ischemia-reperfusion of dogfish shark (*Squalus acanthias*) [77], illustrating the complexity of urea metabolism and function. 

## 4. Materials and Methods

### 4.1. Animal Handling and Ethics

Crucian carp were collected in late summer from Tjernsrud pond in Oslo, Norway, using nylon net cages. Fish were held at the InVivo aquarium facility at the Department of Biosciences, University of Oslo, in tanks with a semi-closed recirculation system in a room with a 12 h cycle of light/dark. The systems were connected to aerated and dechlorinated tap water with temperature around 12 °C. Fish were fed carp pellets (Tetra Pond, Tetra, Melle, Germany) daily, and given at least 2 weeks acclimatization time in the holding tanks prior to any exposures, and fasted for 24 h prior to anoxia exposure. The holding conditions and experimental protocol were approved by the Norwegian Food Safety Authority (FOTS permit ID 16063), in accordance with the animal welfare law of Norway (“Dyrevelferdsloven”) and the instruction about use of animals for research (“Forskrift om bruk av dyr i forsøk”).

### 4.2. Anoxia and Reoxygenation Exposure

Crucian carp (49 g ± 21 g) of mixed sex were placed in 25-L non-transparent plastic containers (except a 5 × 10 cm^2^ window of clear plastic used for daily checks, covered with non-transported plastic sheets), with air-tight lids. Tubing was led out of the top and connected to either an air pump or nitrogen. All tanks had air bubbling for 1–2 days after which the bubbling in the anoxic container was switched to nitrogen. The water temperature was kept constant at 8.25 °C ± 0.25 °C from the point of air bubbling and throughout the entire experiment. Anoxia was achieved by continuous low bubbling of nitrogen, and the oxygen level monitored using Firesting fiber-optic oxygen meter with an oxygen probe (PyroScience GmbH, Aachen, Germany) to ensure that anoxic levels were maintained (<0.1% of air saturation), and kept anoxic for 5 days. 

A total of 64 fish were used, with 16 fish each being exposed to either 5 days normoxia (N), 5 days anoxia (A), 5 days anoxia with 3 h reoxygenation (R3h) or 5 days anoxia with 24 h reoxygenation (R24h). Sampling was performed in a manner that kept the number of fish per exposure tank equal at all times. Of the 64 exposed fish, six fish per treatment (24 fish in total) were included in the present study, and the remaining 40 fish in the exposure experiment were used for another study. Fish were euthanized by a quick blow to the head, and blood was sampled by caudal puncture using a small heparinized syringe after which the spinal cord was cut and brain, heart (ventricle), and liver tissues were dissected out in the mentioned order, wrapped in tin foil and immersed in liquid nitrogen.

### 4.3. Sample Preparation

Blood plasma was separated by mixing the collected blood sample with 13% EDTA-2K solution (blood:EDTA solution = 100:1) in a Venoject^®^ II vacuum blood collection tube. The solution was immediately centrifuged at 1200× *g* for 10 min before 120–150 µL of the blood plasma was dispensed into a new, cold tube and stored at −80 °C until further analysis. Approximately 40 mg tissue of brain, heart, and liver was cut out without thawing and kept at −80 °C until further analysis. Blood plasma and tissue samples of six individual fish from the four experimental groups were shipped to the Human Metabolome technologies (HMT) facility in Yamagata, Japan, for measurement of metabolite concentrations. All samples were kept at −80 °C from the point of collection until analysis.

### 4.4. Metabolomics

Quantitative analysis of 116 target metabolites engaged in central metabolism was performed by HMT (Yamagata, Japan) according to the developed protocols by Soga and colleagues [78,79,80]. Briefly, samples were mixed with 50% acetonitrile in water containing internal standards (10 µM) and homogenized. The supernatant was filtered through a 5 kDa cut-off filter (ULTRAFREE-MC-PLHCC, HMT, Yamagata, Japan) and the remaining filtrate concentrated and resuspended in 50 µL of ultrapure water. Cationic metabolites were analyzed using capillary electrophoresis (CE) time-of-flight (TOF) mass spectrometry (MS) with an Agilent CE-TOFMS system (Agilent technologies), whereas anionic metabolites were analyzed by CE-triple quadrupole (QqQ) MS on an Agilent 6460 TripleQuad LC/MS system (Agilent technologies). CE was performed on fused silica capillary (50 µm × 80 cm) with 30 kV voltage for both cationic and anionic metabolites, whereas an electrospray ionization (ESI) source was connected to both used MS systems. Cationic metabolites were measured in the cation mode with cation running and rinse buffers, positive ESI and an MS capillary voltage set to 4000 V. Anionic metabolites were measured in both positive and negative mode with anionic running and rinse buffers, positive and negative ESI and the MS capillary voltage set to 4500 V. The detected peaks were extracted with automatic integration software for CE-TOFMS (MasterHands ver.2.17.1.11 developed at Keio University) and CE-QqQMS (MassHunter Quantitative Analysis B.06.00 Agilent Technologies, Santa Clara, CA, USA) to obtain *m/z*, migration time and peak area. After converting the peak areas to relative peak areas based on internal standards and a normalization factor, putative metabolites were assigned from the HMT database. The threshold was set to ±0.5 min in migration time and ±10 ppm in *m/z*. Lastly, absolute concentrations of the metabolites were calculated by normalizing the peak area of each metabolite with respect to the peak area of the internal standard by using standard curves obtained by three-point calibrations.

### 4.5. Statistics

Statistical evaluation of the differences in concentrations of each metabolite between the four treatment groups was performed with one-way analysis of variance (ANOVA) with Tukey’s honestly significant difference (HSD) post hoc test. Principal component analysis (PCA) was used to assess overall trends in the data. The data was log2 transformed prior to all analysis, and for PCA plots the data was additionally centered and autoscaled. A *p*-value < 0.05 was considered significant. The PCA plots are statistically based on singular value decomposition. All analyses were performed using the Metaboanalyst 4.0 online software statistics tool [24,25,81]. All graphs were made using Graphpad Prism (GraphPad Prism version 8.0.0 (244) for Windows 64-bit, GraphPad Software, San Diego, CA, USA), and absolute concentrations were plotted, with significances calculated using the same data but log2 transformed to keep results in line with the results of ANOVA from Metaboanalyst. Tukey’s post hoc test was again used. For all statistical analysis, only metabolites that were detected in more than 12 samples (i.e., >50% of the replicates) were included. Missing values (undetected) were set to 0.005 (approximately half the value of the smallest detected metabolite) prior to statistical analysis.

## 5. Conclusions

In this study, we have detected clear differences between both 3 h and 24 h reoxygenation within tissues, and also between tissues, particularly at 3 h reoxygenation. We show that more focus should be given to reoxygenation at different time points in order to reveal the processes that the crucian carp utilize to successfully reoxygenate its tissues while minimizing oxidative damage. Interestingly, the mitochondria of the heart seem to recover quite differently from the other tissues, in terms of the ETC and oxidative phosphorylation during the early reoxygenation stages. This may reflect protective mechanisms but may also be the result of the fact that the heart only receives venous blood that has largely been stripped of oxygen by other tissues, particularly early on during reoxygenation.

The observed high levels of succinate, uric acid and urea in plasma, as well as a high accumulation of succinate and uric acid in the liver and urea in the brain, pose the question of whether or not these metabolites are being transported between tissues. The handling of succinate and uric acid in particular deserves further attention, since it may be indicative of previously undiscovered protective strategies employed by the crucian carp. 

Our data also points to that proteolysis and amino acid metabolism are integral parts of the anaerobic function of crucian carp tissues. Amino acid accumulation in plasma likely suggests transportation of amino acids between tissues, while the sustained elevation of several amino acids during the early stages of reoxygenation may possess protective mechanisms. 

## Data Availability

The data presented in this study are available in the Electronic Supplementary Material.

## Supplementary Materials

The following are available online at https://www.mdpi.com/article/10.3390/metabo11070435/s1, File S1: Raw data for all metabolites as provided by HMT, and *p*-values, FDR and fold changes as calculated using the online MetaboAnalyst software.
